# *LuCSD3* enhances salt stress tolerance in flax: genome-wide profiling and functional validation of the SOD gene family

**DOI:** 10.3389/fpls.2025.1609085

**Published:** 2025-07-03

**Authors:** Yuan Zhang, Ruinan Wang, Hengping Wang, Huiyan Wang

**Affiliations:** ^1^ Jilin Collaborative Innovation Center for Antibody Engineering, Jilin Medical University, Jilin, China; ^2^ College of Laboratory, Jilin Medical University, Jilin, China

**Keywords:** flax, superoxide dismutase, phylogeny analysis, salt stress, reactive oxygen species

## Abstract

Superoxide dismutase (SOD) serves as a critical regulator of plant stress adaptation to salinity, drought, and heavy metal toxicity. Flax (*Linum usitatissimum* L.), a globally cultivated oilseed and fiber crop, lacks comprehensive genomic characterization of its *SOD* gene family. Here, we systematically identified 12 *LuSOD* genes in the flax genome. Phylogenetic reconstruction of SOD homologs across diverse plant species classified these genes into three evolutionarily conserved subgroups: Cu/Zn-SOD (6 *LuCSD*), Fe-SOD (3 *LuFSD*), and Mn-SOD (3 *LuMSD*). Comparative analysis of exon-intron architectures and conserved motifs revealed high structural conservation among *LuSOD* members within each clade. Promoter cis-element profiling identified predominant associations with phytohormone signaling (abscisic acid, methyl jasmonate) and abiotic stress responses, including hypoxia, drought, and low-temperature adaptation. MicroRNA target prediction identified lus-miR159 as the primary regulatory miRNA interacting with *LuSOD* genes. Gene ontology (GO) enrichment highlighted *LuSOD* roles in stress perception, metal ion chelation, and enzymatic reactive oxygen species (ROS) scavenging. Transcriptomic profiling demonstrated ubiquitous high expression of *LuSOD* genes in leaf tissues. qRT-PCR validation under cold, drought, and salt stresses revealed significant upregulation of nine *LuSOD* genes, implicating their involvement in antioxidant defense mechanisms. Functional characterization of *LuCSD3* in transgenic *Arabidopsis* confirmed its role in enhancing salt tolerance through ROS homeostasis modulation. This study provides foundational insights into *LuSOD*-mediated stress resilience, serving as a valuable resource for molecular breeding and functional genomics in flax.

## 1 Introduction

Abiotic stress induced reactive oxygen species (ROS) overproduction in plants, causing oxidative impairment to biomacromolecules, membrane integrity, and cellular ultrastructure, ultimately triggering programmed cell death (Kar, 2011). Superoxide dismutase (SOD), a pivotal enzyme in ROS homeostasis, constituted the first enzymatic barrier against oxidative damage by catalyzing ROS detoxification, thereby safeguarding cellular components from oxidative injury (Abreu and Cabelli, 2010; Gill et al., 2015). Plant SODs were classified into three phylogenetically distinct isoforms—Cu/Zn-SOD, Fe-SOD, and Mn-SOD—based on their metal cofactor specificity. These isoforms exhibited divergent amino acid sequences, subcellular compartmentalization, tertiary structures, and hydrogen peroxide sensitivity profiles (Jiang et al., 2019).

Recent research underscored the pivotal role of Superoxide Dismutases (SODs) in safeguarding plants against diverse abiotic stressors, such as extreme temperatures, drought, salinity, and hormonal fluctuations (Qin, 2023). Certain SOD isoforms operated within specialized cellular compartments to mitigate oxidative stress (Corpas et al., 2017). Cu/Zn-SOD (CSD), the most ubiquitous isoform, localized to chloroplasts, mitochondria, and the cytosol. Mn-SOD (MSD) predominantly resided in mitochondria and peroxisomes, playing essential roles in drought and salinity tolerance (Asensio et al., 2012). Fe-SOD was primarily chloroplast-localized, while Mn-SOD also occurred in peroxisomes. Cu/Zn-SOD additionally occupied extracellular spaces and peroxisomes (Huseynova et al., 2014; Case, 2017). In rape, genomic studies had identified 31 *SOD* genes, with eight exhibiting pronounced upregulation under hormonal and non-biomass stress conditions. Similarly, Salvia miltiorrhiza was found to harbor eight *SOD* genes that displayed distinct responsiveness to cold, salt, drought, heavy metals, and phytohormonal changes (Han et al., 2020). There are 7 members of *SOD* gene family in barley, among which *HvSOD1*, *HvSOD4* and *HvSOD5* expression changed significantly under drought and salt stress (Zhang et al., 2021). In tomatoes, among nine *SlSOD* genes, *SlSOD1* was uniquely upregulated under stress, while *SlSOD2*, *SlSOD5*, *SlSOD6*, and *SlSOD8* responded specifically to salt stress (Feng et al., 2016). Almost all *HbSOD* genes of rubber tree have high expression level under drought stress (Yu et al., 2022). The drought and salt tolerance of overexpressed peanut *AhCu/ZnSOD* in tobacco was significantly higher than that of wild type tobacco, and it could survive for a long time under water shortage (Negi et al., 2015). In tea plants, flavonoid biosynthesis genes and flavonoid levels correlated strongly with SOD activity, a relationship further evidenced in *Arabidopsis*, where *EkFLS* overexpression boosted both flavonoids and *SOD* expression under drought (Wang et al., 2021). Additionally, trehalose was shown to modulate tomato *Cu/ZnSOD* expression during cold stress (Liu et al., 2020). These findings indicate that bolstering *SOD* gene activity and its elevated expression are key in enhancing plant stress resistance.

It has been found that miRNA-mediated ROS transcription regulation plays an important role in improving crop yield and stress resistance (Ravichandran et al., 2019; Cui et al., 2020; Ding et al., 2020). In *Arabidopsis*, two *Cu/ZnSOD* genes are targeted by miR398. There are 20 miRNA targeting 14 *SOD* genes in cotton (Wang et al., 2017). Ghr-miR414c, ghr-miR7267, ghr-miR0081, ghr-miR0166, ghr-miR0362, ghr-miR0362 plays an important role in cotton fiber development (Li et al., 2012). Inhibition of miR398 expression in *Arabidopsis* induced up-regulation of copper/zinc *SOD* gene *CSD1* and *CSD2*, and improved plant antioxidant stress ability (Sunkar et al., 2006). These findings indicate that miRNA plays an important role in environmental signaling and plant development by modifying the *SOD* gene.

Flax (*Linum usitatissimum* L.), a historically significant crop with global cultivation spanning temperate zones, has been utilized for oilseed, fiber, and dual-purpose applications (Huis et al., 2010). Based on its agronomic applications, flax is functionally categorized into three types: oilseed, fiber, and dual-purpose varieties (Chytilova et al., 2013). Flaxseeds are nutritionally dense, containing bioactive compounds such as lignans, dietary fiber, and alpha-linolenic acid (ALA)—an essential omega-3 fatty acid critical for human metabolic and cardiovascular health (Santos et al., 2020). However, in flax cultivation, abiotic stresses such as cold, salinity, and drought severely limit its yield (Yadav et al., 2022). Prior to this study, the *SOD* gene family in flax remained genomically uncharacterized. Here, we systematically investigated the *SOD* gene family in flax using an integrated bioinformatics approach. Analyses encompassed evolutionary relationships, chromosomal distribution, protein physicochemical properties, conserved motifs, promoter cis-elements, protein interaction networks, and tissue-specific expression profiles. Our findings revealed that flax *SOD* genes exhibit transcriptional responsiveness to low-temperature, salinity, and drought stresses, providing mechanistic insights into their roles in stress adaptation. This study represents the first comprehensive genomic characterization of the *SOD* gene family in flax, establishing a foundation for future functional studies aimed at enhancing stress resilience in this economically vital crop.

## 2 Materials and methods

### 2.1 Plant material and statistical analysis

The experiments were conducted using the flax cultivar ‘Longyan 10’ from the Gansu Academy of Agricultural Sciences (Zhang et al., 2020). Seeds were surface-sterilized by immersion in 75% ethanol for 10 minutes, followed by three rinses with sterile deionized water, and subsequently sown into autoclaved nutrient soil. Growth chamber conditions were maintained at 26°C (day)/18°C (night) with a 16-hour photoperiod. Stress treatments were initiated when seedlings reached 6–7 cm in height. For drought simulation, plants were gently uprooted, washed with distilled water to eliminate soil particles, and transferred to hydroponic systems containing 10% (w/v) polyethylene glycol-6000 (PEG-6000). Salt stress was imposed using an identical protocol with 100 mM sodium chloride (NaCl) solution. Control plants were maintained in distilled water, while low-temperature stress groups were incubated at 4°C. Leaf tissues were harvested at 0, 6, 12, and 24 hours post-treatment using synchronous sampling across all groups to eliminate diurnal rhythm interference, with three biological replicates per time point. All samples were flash-frozen in liquid nitrogen and archived at -80°C for subsequent molecular analyses.

All experimental procedures were conducted with a minimum of three biological replicates. Quantitative data are presented as mean ± standard deviation (SD) derived from triplicate measurements. Statistical analyses and graphical representations were performed using GraphPad Prison 8 software (version 8.4.3; GraphPad Software). Significant differences between groups were determined by two-tailed Student’s t-test, with asterisks denoting the following probability thresholds: *P < 0.05, **P < 0.01, ***P < 0.01.

### 2.2 Identification of *SOD* gene in flax

The complete genome assembly of flax (Longya10) was retrieved from the NCBI database under accession number QMEI02000000, while genome annotation files were sourced from the Figshare repository (https://figshare.com/articles/dataset/Annotation_files_for_Longya-10_genome/13614311). Eight *Arabidopsis SOD* homologs were analyzed via the TAIR database (Garcia-Hernandez et al., 2002). Candidate flax *SOD* genes were identified through BLASTP homology searches against the flax proteome (E-value cutoff: 1e-5). Hidden Markov Models (HMMs) for Cu/Zn-SOD (PF00080) and Fe/Mn-SOD (PF02777, PF00081) domains were acquired from the Pfam database (http://pfam.xfam.org/) (Mistry et al., 2021). The HMMER3.0 hmmsearch algorithm (Potter et al., 2018) was employed to predict *SOD* homologs in flax. Predicted sequences were further validated using SMART (http://smart.embl.de/smart/batch.pl) and the Conserved Domain Database (CDD; https://www.ncbi.nlm.nih.gov/cdd/), yielding a final set of 12 non-redundant *LuSOD* genes. Biophysical parameters of the *LuSOD* proteins—including coding sequence length, amino acid count, molecular weight (MW), theoretical isoelectric point (pI), and grand average of hydropathy (GRAVY)—were computationally derived using ExPASy ProtParam (https://web.expasy.org/protparam/) (Fink and Scandalios, 2002). Subcellular localization predictions were performed via the BUSCA web server (http://www.busca.cn).

### 2.3 Phylogeny, chromosome location, conserved domain and conserved motif of *LuSOD* gene

The full-length genomic sequences of rice and soybean were acquired from the Phytozome platform (https://phytozome-next.jgi.doe.gov/) (Goodstein et al., 2012). *SOD* gene families in these species were characterized using identical bioinformatics workflows. Multiple sequence alignment of *SOD* proteins from *Arabidopsis*, rice, soybean, and flax was performed using ClustalW in MEGA11 with default parameters (Yuan et al., 1999). A maximum likelihood (ML)-based phylogenetic tree was generated in MEGA11 under standard configurations (neighbor-joining algorithm; 1,000 bootstrap replicates) (Tamura et al., 2021). Chromosomal localization of *LuSOD* genes was mapped by integrating the flax genomic FASTA file with GFF3 annotations. Conserved structural domains were predicted via the NCBI CD-Search tool (https://www.ncbi.nlm.nih.gov/Structure/BWRPSB/BWRPSB.cgi). Conserved protein motifs were identified using MEME Suite (http://alternate.meme-suite.org/tools/meme), and visualization was executed with TBtools v2.069 (Chen et al., 2020).

### 2.4 Genome-wide replication and collinear analysis of *LuSOD* gene

The genome and annotation files of *Arabidopsis* were retrieved from the TAIR database (https://www.arabidopsis.org/). Collinear relationships were predicted using the Multiple Collinearity Scan (MCScanX) algorithm (Wang et al., 2012). Genome-wide duplication (WGD) events involving *LuSOD* genes were identified through whole-genome synteny analysis. Tandem duplication events were defined as chromosomal regions harboring two or more homologous genes within a 100-kb span, with no intervening non-homologous genes. Segmental duplication events (BLASTN E-value < 1e-5) were detected by analyzing 100-kb genomic regions (50 kb upstream and downstream) flanking coding sequences (CDS) using BLASTN alignments. Repetitive genes were classified based on sequence alignment length ≥200 base pairs (bp) and nucleotide sequence identity exceeding 85% (Khaja et al., 2006).

### 2.5 MiRNA prediction and cis-acting element analysis

Potential miRNA targets of the *LuSOD* gene family were predicted by aligning miRNA sequences with the 5’ and 3’ untranslated regions (UTRs) and coding sequences (CDS) using the psRNATarget platform (Plant MicroRNA Target Analysis Server; https://www.zhaolab.org/psRNATarget/analysis?function=3) under default parameters (Griffiths-Jones et al., 2006). The 2,000-bp upstream promoter regions of *LuSOD* genes were isolated from the flax genome using the TBtools software suite. To elucidate the transcriptional regulatory mechanisms of *LuSOD* genes under environmental stress, cis-acting elements within the 2.0-kb promoter sequences upstream of the translation start site were annotated via the PlantCARE database (http://bioinformatics.psb.ugent.be/webtools/plantcare/html/) (Lescot et al., 2002). Identified motifs were visualized using TBtools (version 2.069) for comparative analysis.

### 2.6 Construction of protein interaction network and GO enrichment analysis

To delineate the protein interaction network of the *LuSOD* gene family, orthologous *Arabidopsis SOD* genes were employed as reference sequences. Functional protein-protein interaction (PPI) networks were reconstructed using the STRING database (v11.5; https://string-db.org/) under standard configurations (Franceschini et al., 2013). For Gene Ontology (GO) enrichment analysis, the flax proteome was annotated via the eggNOG-mapper platform (http://eggnog-mapper.embl.de/) using the GO-base.ob reference file integrated within TBtools, yielding comprehensive functional annotations (Cantalapiedra et al., 2021). Resultant datasets were visualized using TBtools.

### 2.7 Expression pattern analysis of the *LuSOD* gene family and construction of *LuCSD3*-transgenic *Arabidopsis*

In this study, five flax transcriptional groups were sequenced: (a) pistil, stamen, fruit and stem tip tissues (PRJNA1002756) (https://www.ncbi.nlm.nih.gov/sra/?term=); (b) floral tissues at 30, 20, 10 and 5 days after anthesis (PRJNA833557); (c) different flax embryo tissues, anther and seed tissues (PRJNA663265); (d) root and leaf tissues after salt stress (PRJNA977728) (e) Stem tissue (PRJNA874329) after heat stress. The data were filtered by fastp, then compared to the Longya10 reference genome (Chen et al., 2018). Transcript abundance was quantified as FPKM (Fragments Per Kilobase Million), and log2-transformed FPKM values were visualized as clustered heatmaps using TBtools.

Transgenic *Arabidopsis* was obtained via the floral dip method (Clough and Bent, 1998). The *Agrobacterium* harboring the intact 35S::*LuCSD3* construct was inoculated into 150 mL of LB liquid medium and cultured at 200 rpm and 28°C for 16 hours. Cells were collected by centrifugation at 5000 rpm for 10 minutes and resuspended in a 5% sucrose solution adjusted to OD600 = 1.0, supplemented with 0.01% Silwet-77 surfactant. Flower buds at the pre-bolting stage were immersed in the transformation solution for 2 minutes, with three rounds of infection per week. T1 generation plants were obtained and screened. T2 lines that produced 100% hygromycin-resistant plants in the T3 generation were identified as homozygous transgenic lines.

### 2.8 RNA extraction and fluorescence quantitative PCR analysis

Total RNA was isolated from flax leaf tissues using the Trizol reagent-based protocol. The SPARKscript II RT Plus Kit (With gDNA Eraser) (Shandong Sparkjade Biotechnology Co., Ltd.) was utilised to create cDNA. Gene-specific primers (**Supplementary Table S5**) were designed using Oligo 7 primer design software, and quantitative real-time PCR amplification was conducted with TB Green^®^ Premix Ex Taq™ II. Relative gene expression levels were calculated via the 2^−ΔΔCT^ method (Livak and Schmittgen, 2001), with GAPDH serving as the internal reference gene. Three technical replicates were analyzed per sample to determine cycle threshold (Ct) values.

### 2.9 Phenotypic analysis and NBT staining experiment of *Arabidopsis* with *LuCSD3* gene transfer

To assess the salt stress tolerance phenotypes of T3 transgenic *Arabidopsis*, two-week-old wild-type (Col-0) and overexpression lines (OE) were divided into treatment and control groups. The treatment cohort was subjected to 200 mM NaCl irrigation every three days for 15 days, while controls received equivalent volumes of distilled water. Following the stress regimen, the third fully expanded rosette leaves from both groups were harvested for nitroblue tetrazolium (NBT) staining. The NBT working solution was formulated by dissolving 0.05 g NBT powder in 0.5 mL phosphate-buffered solution (PBS, pH 7.8), followed by dilution to 50 mL with deionized water. Leaf samples were incubated in the NBT solution for 30–60 minutes under dark conditions. Subsequently, stained leaves were destained in 95% ethanol until complete chlorophyll removal for histological observation. All experimental procedures were performed with three biological replicates to ensure statistical robustness.

### 2.10 Physiological and biochemical indicators detection of transgenic *Arabidopsis* with *LuCSD3* gene

The physiological and biochemical indicators of the control group and OE lines (OE-1 and OE-5) were measured after 15 days of 200 mM NaCl stress. The parameters included malondialdehyde (MDA) content, proline (Pro) content, superoxide dismutase (SOD) activity, peroxidase (POD) activity, and hydrogen peroxide (H_2_O_2_) content. All reagent kits were provided by Beijing Boxbio Science & Technology Co.,Ltd. The measurements were conducted following the methods described in previous studies (Lu et al., 2025). Each sample was analyzed in triplicate.

## 3 Results

### 3.1 Identification and phylogenetic analysis of *SOD* gene family in flax

**Figure 1:**
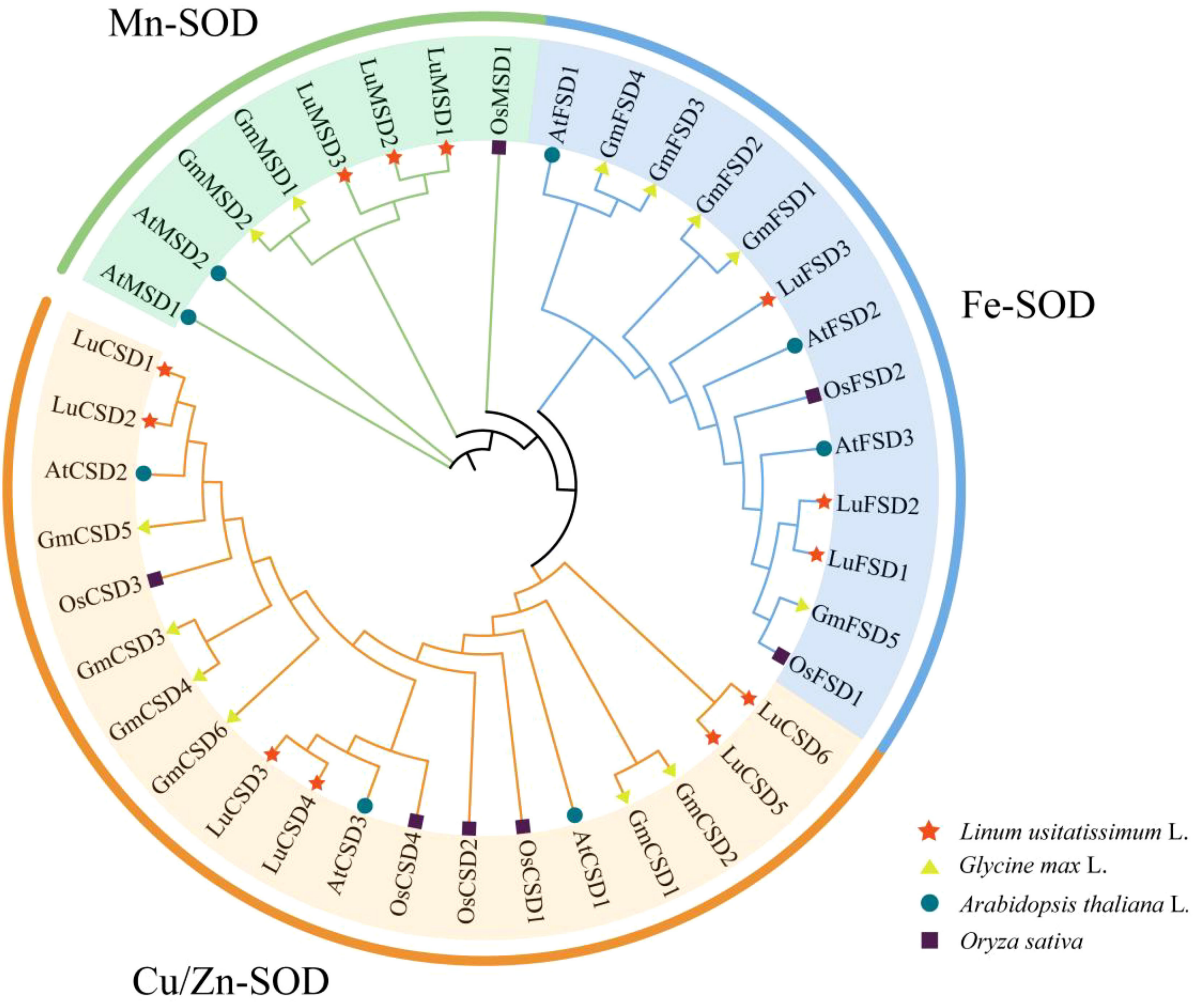
*SOD* protein phylogenetic tree of four species. The pentagram represents the flax gene, the circle represents the *Arabidopsis* gene, the triangle represents the soybean, and the square represents the rice gene. All *SOD* genes can be divided into three subfamilies: Cu/Zn-SOD, Mn-SOD and Fe-SOD, which are represented by different colors.

Utilizing Hidden Markov Model (HMM) profiles of Cu/ZnSOD (PF00080) and Fe/MnSOD (PF02777, PF00081), 12 *SOD* genes were identified in the flax (Longya10) genome. Phylogenetic orthology with *Arabidopsis SOD* homologs led to their nomenclature as *LuCSD1*–*LuCSD6* (Cu/Zn-SOD), *LuFSD1*–*LuFSD3* (Fe-SOD), and *LuMSD1*–*LuMSD3* (Mn-SOD) (**Table 1**; **Figure 1**). Biochemical characterization of the encoded proteins revealed substantial variation: *LuMSD1* encoded the longest polypeptide (384 amino acids), while *LuCSD5* represented the shortest (152 amino acids). Molecular weights of *LuSOD* proteins spanned 15.35–42.36 kDa. Isoelectric point (pI) analysis indicated that only four proteins (*LuCSD3*, *LuMSD1*, *LuMSD2*, and *LuMSD3*) exhibited pI values >7, suggesting a predominance of acidic amino acids in the *LuSOD* family. Instability indices ranged from 15.74 (stable) to 51.0 (unstable), with all *LuSOD* proteins except *LuFSD3* classified as stable. Aliphatic indices varied between 72.71 and 91.04, reflecting differences in thermostability. Hydropathicity analysis predicted *LuCSD2* as the sole hydrophobic protein, whereas others were hydrophilic. Subcellular localization predictions assigned five genes (*LuFSD1*, *LuFSD2*, *LuMSD1*–*LuMSD3*) to mitochondria, with remaining members localized to chloroplasts.

**Table 1:** Prediction and characterization of *SOD* Gene in Flax.

Gene	Gene ID in Genome	Number of Amino Acids	Molecular Weight (kDa)	PI	Instability Index	Aliphatic Index	Grand Average of Hydropathicity (GRAVY)	Subcellular Localization
*LuCSD1*	L.us.o.m.scaffold131.124	220	22.39	5.95	22.11	86.50	-0.006	Chloroplast
*LuCSD2*	L.us.o.m.scaffold262.55	222	22.51	5.95	20.27	91.04	0.076	Chloroplast
*LuCSD3*	L.us.o.m.scaffold141.48	299	30.86	7.75	28.41	76.96	-0.252	Chloroplast
*LuCSD4*	L.us.o.m.scaffold6.145	361	38.32	6.2	35.56	72.71	-0.194	Chloroplast
*LuCSD5*	L.us.o.m.scaffold77.257	152	15.35	5.70	15.74	76.32	-0.168	Chloroplast
*LuCSD6*	L.us.o.m.scaffold382.12	254	26.54	6.69	36.59	82.52	-0.169	Chloroplast
*LuFSD1*	L.us.o.m.scaffold15.135	273	31.28	6.14	33.67	78.64	-0.292	Mitochondrion
*LuFSD2*	L.us.o.m.scaffold76.116	274	31.23	6.14	33.52	79.78	-0.330	Mitochondrion
*LuFSD3*	L.us.o.m.scaffold6.395	281	32.2	5.62	51.00	78.86	-0.534	Chloroplast
*LuMSD1*	L.us.o.m.scaffold67.11	384	42.36	9.91	48.46	80.78	-0.421	Mitochondrion
*LuMSD2*	L.us.o.m.scaffold34.265	379	41.86	10.01	46.89	80.05	-0.455	Mitochondrion
*LuMSD3*	L.us.o.m.scaffold31.418	246	26.96	7.89	38.81	88.05	-0.262	Mitochondrion

**Figure 2:**
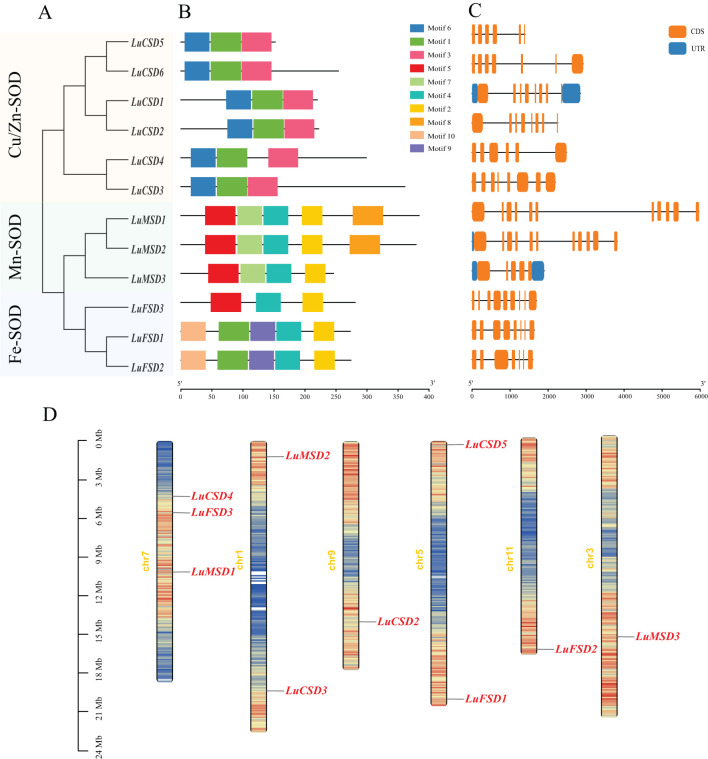
Structural and genomic characterization of the *LuSOD* gene family. **(A)** Phylogenetic clustering of *LuSOD* genes. **(B)** Conserved motif distribution, with gray lines indicating gene length. **(C)** Exon-intron architectures, highlighting structural conservation within subfamilies. **(D)** Chromosomal localization, visualized with a 100-kb sliding window; base density gradients are color-mapped from red (highest) to blue (lowest).

To resolve evolutionary relationships, a phylogenetic tree was reconstructed from 40 *SOD* protein sequences across *Arabidopsis* (8), *Glycine max* (13), *Oryza sativa* (7), and *Linum usitatissimum* (12) (**Figure 2**). According to *Arabidopsis SOD* protein subfamily classification (**Supplementary Table S1**), 12 *SOD* genes in flax were divided into three subfamilies, named Mn-SOD, Fe-SOD and Cu/Zn-SOD respectively. Twelve *LuSOD* genes were distributed in each subfamily, and the largest number of genes was in the Cu/Zn-SOD subfamily, including 6 members (*LuCSD1*-*LuCSD6*). Further studies showed that there was a close relationship between flax and *SOD* family of *Arabidopsis* among the three middle species.

### 3.2 Analysis of gene structure and conservative motif of *LuSOD*

To characterize the protein structural features of the flax *SOD* gene family, the amino acid sequences of 12 *LuSOD* homologs were subjected to conserved motif analysis using the MEME online platform. Ten evolutionarily conserved motifs (annotated as Motif 1–Motif 10) were systematically identified across the *LuSOD* protein sequences (**Figures 2A, B**; **Supplementary Table S2**). *LuCSD* gene contains only three domains (motif1, motif3 and motif6), among which motif3 and motif6 are unique domains of *LuCSD* gene. We also found that both *LuMSD* and *LuFSD* genes contain motif2 and motif4, in which motif7 and motif8 are unique domains of *LuMSD* gene. Among *LuFSD1* and *LuFSD2* genes, motif9 and motif10 are their unique domains. There are great differences in protein domains among different subfamilies of *LuSOD* genes, but the same subfamilies have the same domain, which also proves that different subfamilies have different biological functions.

The analysis of the structure of *LuSOD* gene shows that the number of exons is between 5 and 11, and the number of introns is between 4 and 10 (**Figure 2C**). Both *LuMSD1* and *LuMSD2* contain 11 exons and 10 introns, four genes (*LuCSD1*, *LuCSD2*, *LuCSD3* and *LuFSD3*) contain 8 exons and 7 introns, two genes (*LuCSD6* and *LuFSD2*) contain 7 exons and 6 introns, *LuCSD5* and *LuCSD4* both contain 6 exons and 5 introns. The least number of exons and introns in LuMSD3 are 5 and 4 respectively.

### 3.3 Chromosome mapping and collinearity analysis of *LuSOD* gene

**Figure 3:**
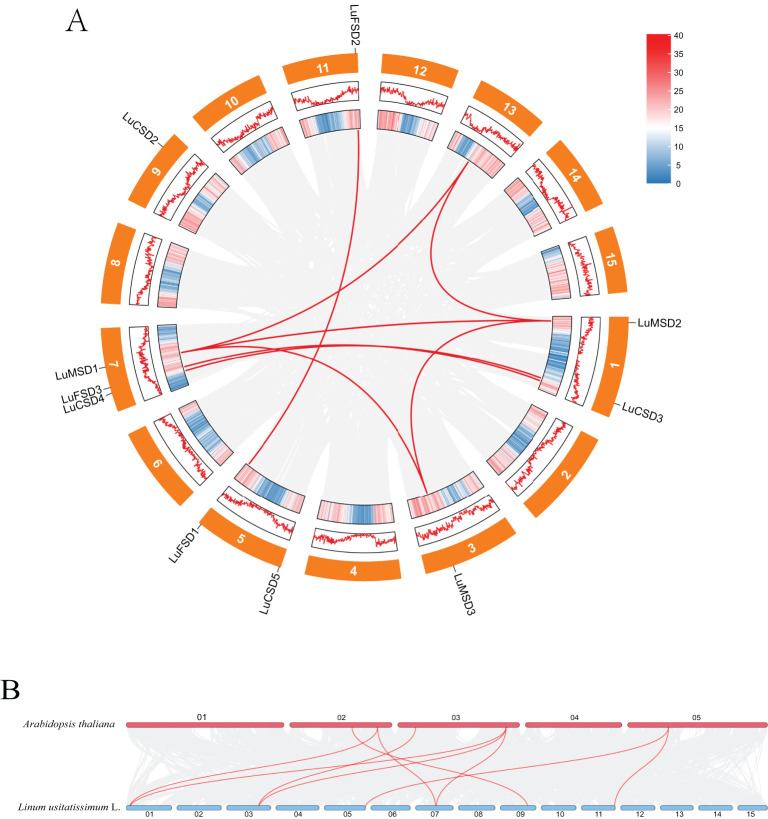
Syntenic relationships of *LuSOD* genes. **(A)** Intra-genomic collinearity among *LuSOD* loci. **(B)** Cross-species collinearity between flax and *Arabidopsis SOD* genes. Gray lines denote genome-wide syntenic blocks, while red lines highlight *LuSOD*-specific orthologous pairs.

Chromosomal localization of the *LuSOD* gene family was mapped using the flax reference genome, revealing an uneven distribution of 12 *LuSOD* genes across six chromosomes (**Figure 2D**). Chromosome 7 harbored the highest number of *LuSOD* genes (3 genes, 25% of the total), followed by chromosomes 1 and 5 (2 genes each, 16.67%), while chromosomes 3, 9, and 11 each contained a single *LuSOD* locus (8.33% per chromosome). To investigate duplication events, a collinearity analysis of the *LuSOD* family was performed via Circos visualization (**Figure 3A**), identifying eight segmental duplication pairs, indicative of substantial gene family expansion. To elucidate evolutionary conservation, syntenic relationships between flax and *Arabidopsis SOD* homologs were analyzed (**Figure 3B**). Nine collinear ortholog pairs were identified, with flax chromosomes 1, 3, 5, 7, 9, and 11 exhibiting synteny to *Arabidopsis* chromosomes 2, 3, and 5. Notably, no collinear *SOD* gene pairs were detected on *Arabidopsis* chromosomes 1 and 4. These findings collectively demonstrate strong chromosomal conservation of *SOD* genes between flax and *Arabidopsis*, with lineage-specific divergence in genomic organization.

### 3.4 *LuSOD* regulatory element profiling and functional network analysis

**Figure 4:**
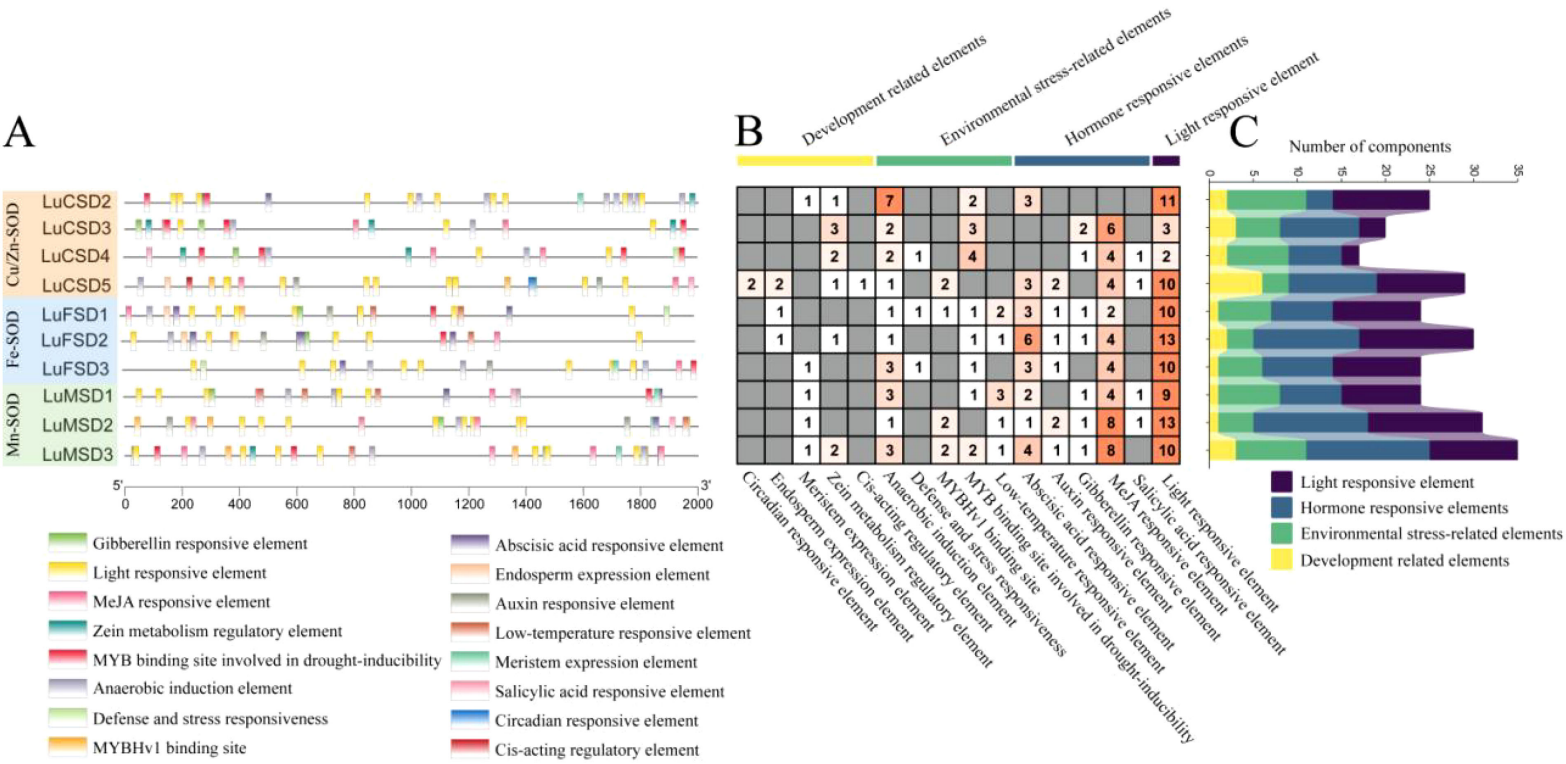
Cis-acting regulatory element profiling of *LuSOD* promoters. **(A)** Subfamily-specific distribution of cis-elements. **(B, C)** Quantitative analysis of motifs associated with developmental regulation (yellow), environmental stress (green), hormonal signaling (dark blue), and light response (purple).

To elucidate the regulatory mechanisms of the *LuSOD* gene family under abiotic stress, promoter regions spanning 2000 bp upstream of the *LuSOD* loci were analyzed and visualized (**Figure 4**; **Supplementary Table S3**). Due to genomic proximity, the upstream sequences of *LuCSD1* and *LuCSD6* could not be isolated, likely resulting from overlapping or truncated intergenic regions. Consequently, promoter analyses focused on the remaining 10 *LuSOD* genes, excluding ubiquitous elements such as CAAT-box and TATA-box. A total of 259 cis-regulatory elements were identified and categorized into four functional groups: developmental regulation, environmental stress adaptation, phytohormone signaling, and light responsiveness (**Figure 4**). Light-responsive elements constituted the predominant category (91 elements, 35.14%), with conserved motifs including G-box, I-box, and Box 4. The second largest group comprised hormone-related elements (89 elements, 34.36%), dominated by methyl jasmonate (MeJA)-responsive motifs (TGACG and CGTCA), alongside abscisic acid (ABRE), auxin (TGA-element, AuxRR-core), gibberellin (GARE-motif), and salicylic acid (TCA-element) response elements. Notably, MeJA-associated motifs were the most abundant hormonal regulators in *LuSOD* promoters. Environmental stress-responsive elements (57 elements, 22.01%) included anaerobic induction (ARE), drought-inducible MYB binding sites (MBS), low-temperature response (LTR), MYBHv1 recognition sites (CCAAT-box), and defense/stress-related TC-rich repeats. Developmental elements (22 elements, 8.5%) encompassed zein metabolism regulators (O2-site), meristem-specific motifs (CAT-box), and endosperm activity markers (GCN4_motif).

MiRNA prediction results showed that among the 12 *LuSOD* gene families, only 7 family members (58.33%) predicted 17 miRNA targets (**Table 2**). Among them, *LuCSD1* gene predicted the most miRNA targets, including lus-miR159b/c and lus-miR398b/c/d/e. *LuFSD2* gene has the least target and contains only one miRNA target (lus-miR828a). We found that the same *LuSOD* gene can be targeted by different miRNA. For example, *LuCSD1* can be targeted by both lus-miR159 and lus-miR398, and *LuMSD2* gene can be targeted by lus-miR156 and lus-miR530 at the same time. We also found that different *LuSOD* genes can be targeted by the same miRNA. For example, lus-miR319 can target both *LuCSD3* and *LuCSD4* genes, lus-miR156 can target both *LuMSD1* and *LuMSD2* genes, and lus-miR159 can target *LuCSD1*, *LuCSD3* and *LuCSD4* at the same time. The results show that lus-miR159 is the main target miRNA of *LuSOD* gene family.light response elements.

**Table 2:** Potential miRNA targets of *LuSOD* gene.

MiRNA	Target	Expectation	MiRNA Length	Target_start	Target_end	Inhibition	Multiplicity
lus-miR828a	LuFSD2	5	20	209	229	Translation	1
lus-miR319a	LuCSD3	5	20	206	226	Cleavage	1
lus-miR319a	LuCSD4	5	20	206	226	Cleavage	1
lus-miR319b	LuCSD3	5	19	207	226	Cleavage	1
lus-miR319b	LuCSD4	5	19	207	226	Cleavage	1
lus-miR159b/c	LuCSD1	4.5	20	378	398	Cleavage	1
lus-miR171a/f	LuMSD3	4.5	20	504	524	Cleavage	1
lus-miR156a/g	LuMSD1	5	19	7	26	Cleavage	1
lus-miR156a/g	LuMSD2	5	19	7	26	Cleavage	1
lus-miR159b/c	LuCSD3	5	20	207	227	Cleavage	1
lus-miR159b/c	LuCSD4	5	20	207	227	Cleavage	1
lus-miR398b/c	LuCSD1	5	20	439	459	Cleavage	1
lus-miR398d/e	LuCSD1	5	21	438	459	Cleavage	1
lus-miR530a/b	LuMSD2	5	19	940	959	Cleavage	1

**Figure 5:**
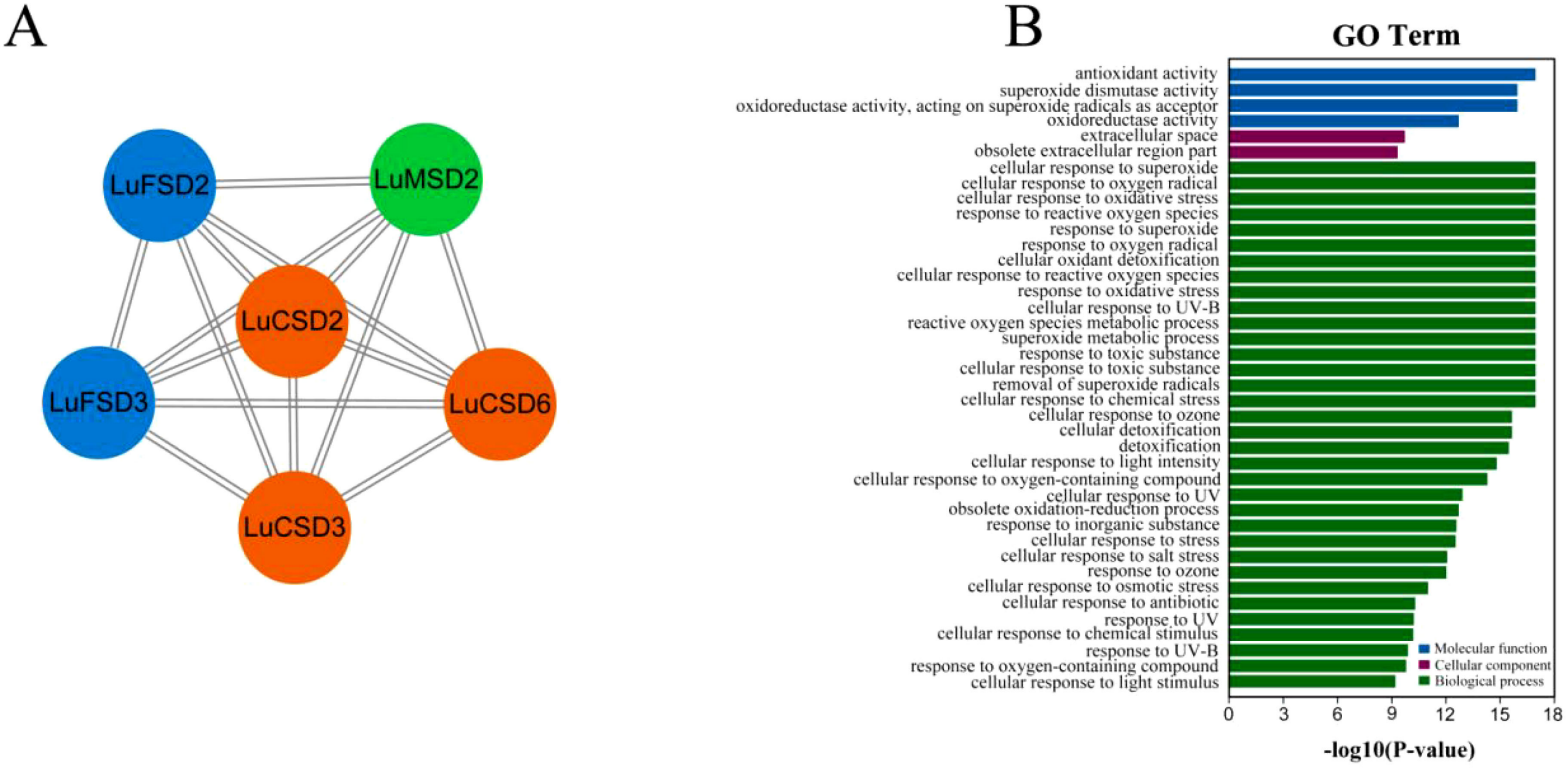
Protein interaction networks and functional annotation of *LuSOD* genes. **(A)** Predicted protein-protein interaction (PPI) networks based on *Arabidopsis* homologs, color-coded by subfamily: *LuCSD* (orange), *LuMSD* (green), and *LuFSD* (blue). **(B)** GO enrichment, categorized into molecular function (blue), cellular component (purple), and biological process (green).

To investigate functional linkages between flax *SOD* proteins and their regulatory roles, a protein-protein interaction (PPI) network was constructed using *Arabidopsis* homologs as a reference framework (**Figure 5A**). The analysis revealed pairwise interactions among six *LuSOD* genes (*LuCSD2*, *LuCSD3*, *LuCSD6*, *LuFSD2*, *LuFSD3*, and *LuMSD2*), whereas the remaining six genes showed no connectivity. The strong evolutionary conservation between *LuSOD* and *AtSOD* proteins suggested functional parallels, particularly given the established role of *Arabidopsis SODs* in mitigating biotic and abiotic stressors. These findings implied that the six interacting *LuSOD* genes may mediate analogous stress-responsive mechanisms in flax.

Gene Ontology (GO) enrichment analysis further delineated the functional spectrum of *LuSOD* genes across three domains: molecular functions, cellular components, and biological processes (**Figure 5B**; **Supplementary Table S4**). Molecular functions were primarily associated with antioxidant activity and superoxide dismutase activity. Cellular component annotations highlighted localization to the extracellular space and archived extracellular regions (obsolete classification). Biological processes predominantly involved cellular responses to superoxide radicals, oxygen radical detoxification, and oxidative stress adaptation, underscoring the pivotal role of *LuSOD* genes in stress resilience.

### 3.5 *LuSOD* expression patterns and abiotic stress responses in flax

**Figure 6:**
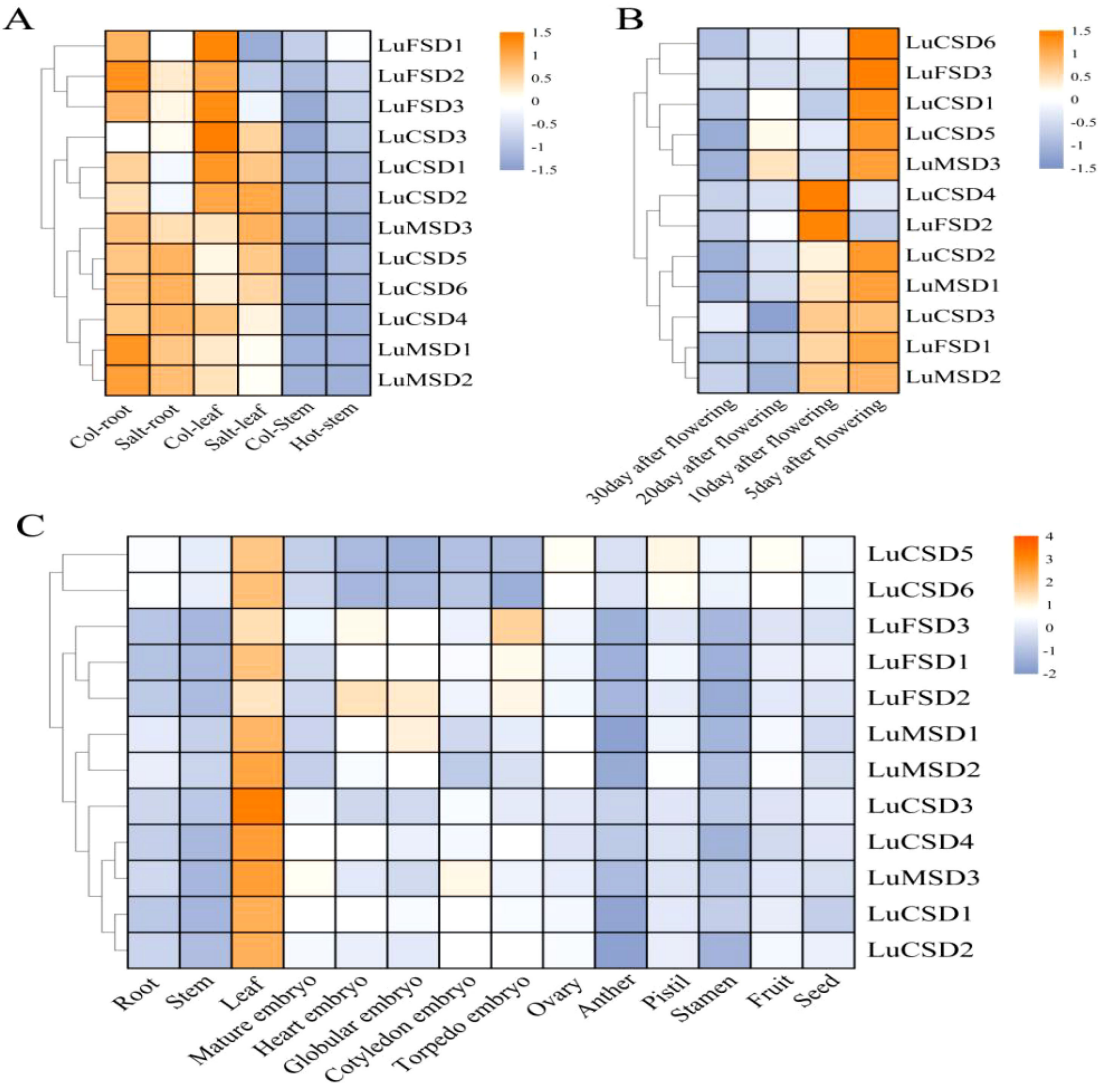
Spatiotemporal expression dynamics of *LuSOD* genes. **(A)** Transcriptional responses to salt and heat stress. **(B)** Expression profiles in post-anthesis floral tissues. **(C)** Tissue-specific expression patterns across flax organs. Expression levels are normalized as log2-transformed FPKM values, color-scaled from high (orange) to low (blue-purple).

Transcriptomic analysis of the *LuSOD* gene family under abiotic stress revealed distinct expression dynamics: under heat stress, only *LuFSD1* exhibited elevated expression in stems, while salt stress significantly suppressed most *LuSOD* genes in leaves and stems (**Figure 6A**). However, four genes (*LuCSD3*, *LuCSD4*, *LuCSD5*, and *LuCSD6*) were upregulated in roots, and three (*LuMSD3*, *LuCSD5*, and *LuCSD6*) showed marked induction in leaves under salt stress, with the *LuFSD* subfamily displaying pronounced sensitivity to salt-induced repression. Tissue-specific expression profiling further uncovered spatiotemporal regulation: in floral tissues (**Figures 6B, C**), all *LuSOD* genes except *LuCSD4* and *LuFSD2* were highly expressed at 5 days post-anthesis (DPA), followed by seven genes (*LuCSD4*, *LuFSD2*, *LuCSD2*, *LuMSD1*, *LuCSD3*, *LuFSD1*, and *LuMSD2*) at 10 DPA, three (*LuCSD1*, *LuCSD5*, and *LuMSD3*) at 20 DPA, and universal downregulation by 30 DPA. Vegetative tissues showed dominant *LuSOD* expression in leaves (notably *LuCSD3*), contrasting with minimal activity in roots, stems, anthers, stamens, and seeds. Embryo development stages revealed specialized roles: *LuMSD3* peaked in mature and cotyledon-stage embryos; *LuFSD3* and *LuFSD2* in heart-stage embryos; *LuFSD2* and *LuMSD1* in globular embryos; and the *LuFSD* subfamily in torpedo-stage embryos. Reproductive organs exhibited coordinated upregulation of *LuCSD5* and *LuCSD6* in ovaries, pistils, and fruits. Collectively, the robust expression of *LuSOD* genes, particularly *LuCSD3*, in leaf tissues underscored their pivotal role in maintaining ROS homeostasis, positioning leaves as central hubs for antioxidant defense in flax.

**Figure 7:**
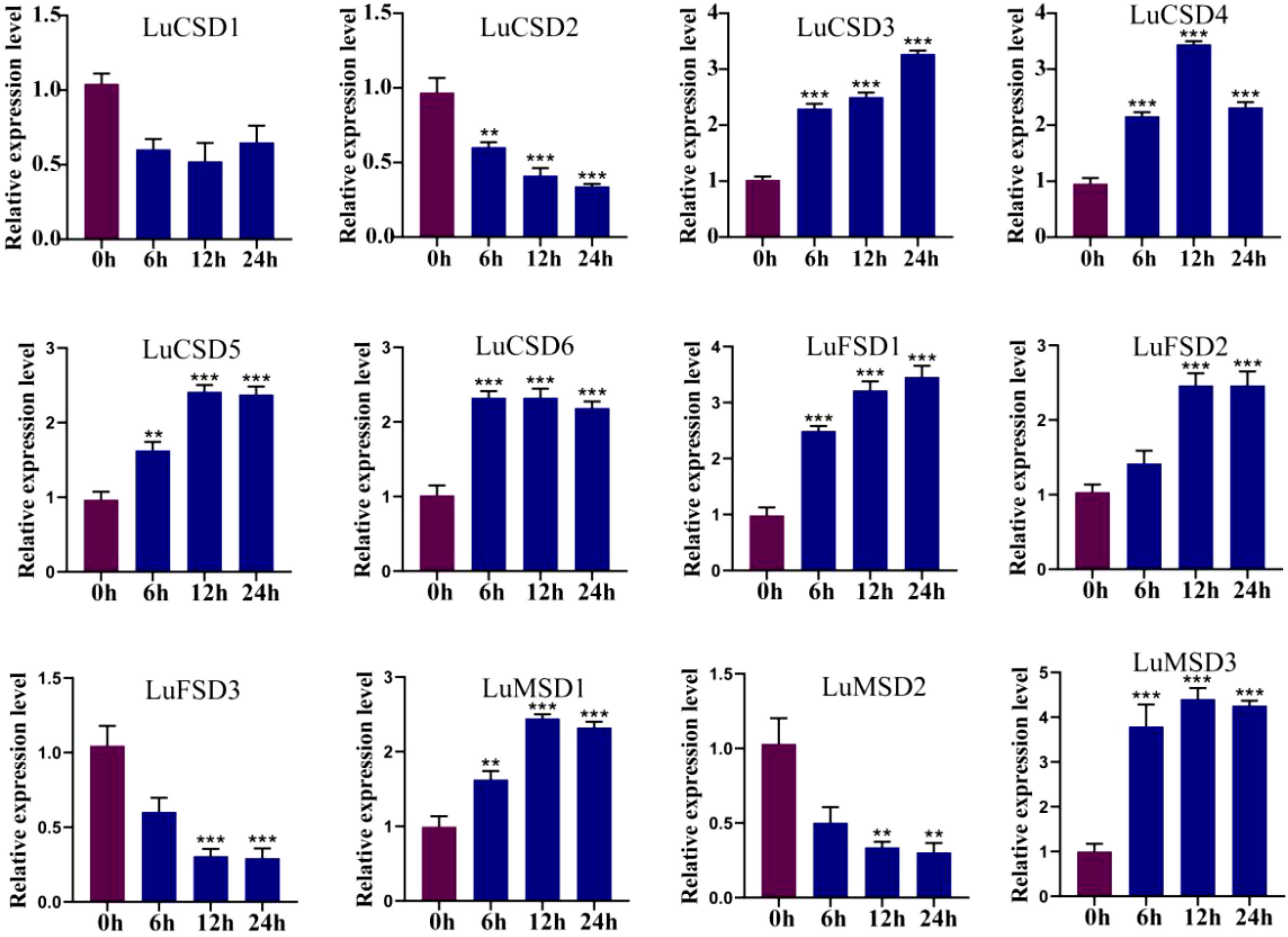
*LuSOD* gene expression under salt stress. Dark purple and dark blue bars represent untreated controls and salt-stressed samples, respectively. Asterisks denote statistically significant differences (Student’s t-test: **p < 0.01; ***p < 0.001).

**Figure 8:**
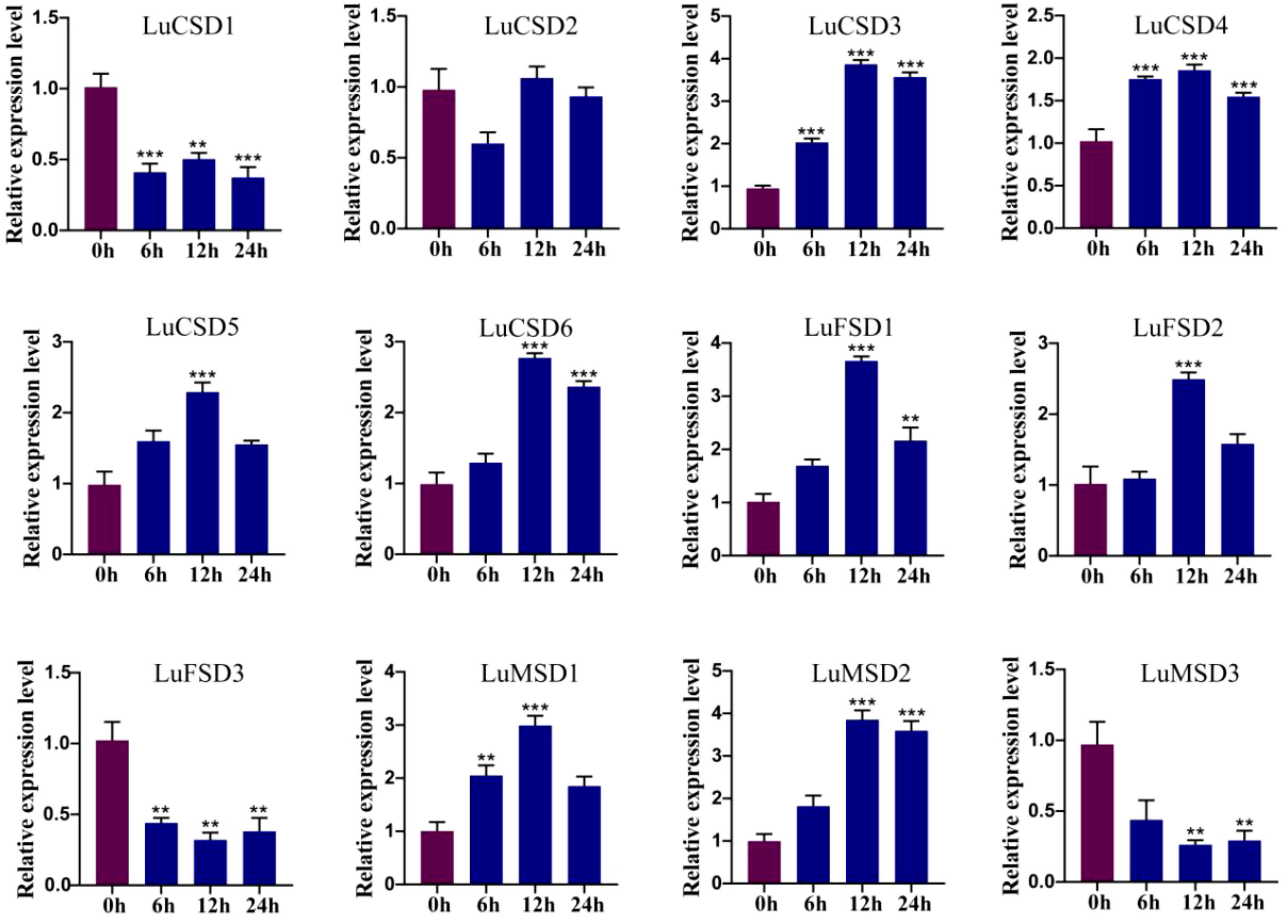
*LuSOD* gene expression under cold stress. Dark purple and dark blue bars represent untreated controls and salt-stressed samples, respectively. Asterisks denote statistically significant differences (Student’s t-test: **p < 0.01; ***p < 0.001).

**Figure 9:**
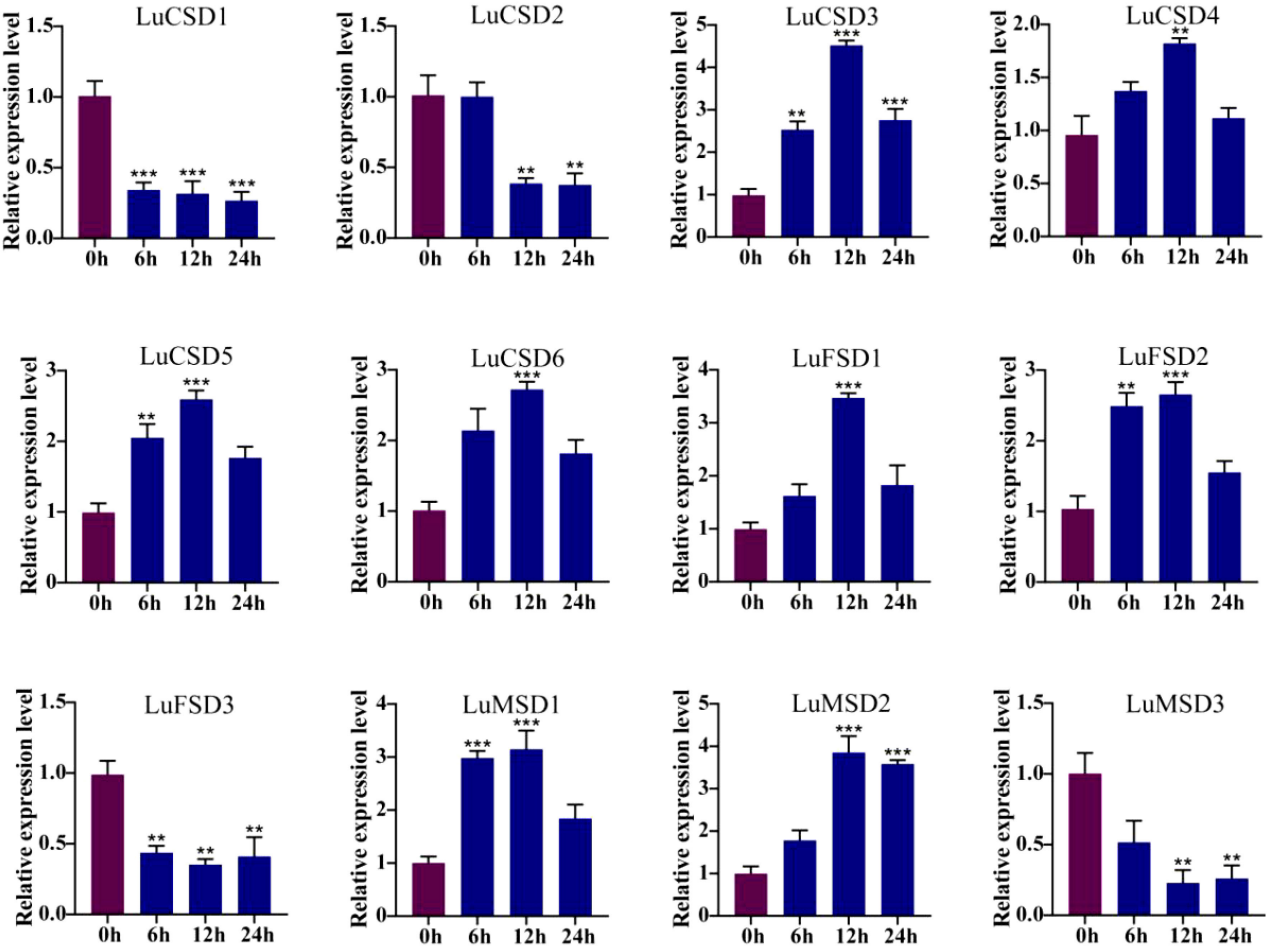
*LuSOD* gene expression under drought stress. Dark purple and dark blue bars represent untreated controls and salt-stressed samples, respectively. Asterisks denote statistically significant differences (Student’s t-test: **p < 0.01; ***p < 0.001).

To evaluate the involvement of *LuSOD* genes in abiotic stress responses (salt, cold, drought), qRT-PCR was employed to quantify their relative expression levels in flax leaf tissues under salt stress. Transcriptional dynamics were assessed at 0, 6, 12, and 24 hours post-stress induction, with expression levels normalized to the 0-hour control (**Figures 7**–**9**). Salt stress triggered time-dependent transcriptional reprogramming within the *LuSOD* family (**Figure 7**). Six genes (*LuCSD4*, *LuCSD5*, *LuCSD6*, *LuFSD2*, *LuMSD1*, and *LuMSD3*) displayed pronounced upregulation under 12-hour salt stress, peaking at 2.3-, 1.3-, 1.25-, 2.4-, and 3.1-fold increases relative to controls, respectively. Two genes (*LuCSD3* and *LuFSD1*) exhibited maximal induction (2.1- and 2.3-fold increases) after 24 hours of salt exposure. In contrast, *LuCSD1* showed no transcriptional response to salt stress in leaves. Conversely, three genes (*LuCSD2*, *LuFSD3*, and *LuMSD2*) demonstrated substantial downregulation, reaching minimal expression levels at 24 hours.

Under prolonged abiotic stress conditions, the *LuSOD* gene family exhibited dynamic transcriptional regulation in flax leaf tissues. During cold stress (**Figure 8**), eight genes (*LuCSD3*, *LuCSD4*, *LuCSD5*, *LuCSD6*, *LuFSD1*, *LuFSD2*, *LuMSD1*, and *LuMSD2*) showed pronounced upregulation at 12 hours, peaking at 2.8-, 0.8-, 1.2-, 1.6-, 2.5-, 1.4-, 1.9-, and 2.8-fold induction, respectively, followed by gradual attenuation, while *LuCSD2* remained unresponsive. Three genes (*LuCSD1*, *LuFSD3*, and *LuMSD3*) were significantly downregulated, reaching minimal expression levels at 24 hours, suggesting their potential role in cold adaptation through negative regulatory mechanisms. Similarly, under drought stress (**Figure 9**), the same eight genes displayed marked upregulation at 12 hours, with peak expression levels of 3.3-, 0.7-, 1.4-, 1.5-, 2.4-, 1.5-, 2.1-, and 2.8-fold increases, respectively, whereas *LuCSD1*, *LuFSD3*, and *LuMSD3* exhibited sustained downregulation, implicating their involvement in drought-responsive suppression pathways. Notably, *LuCSD3* consistently demonstrated robust upregulation under both stress conditions, highlighting its central role in stress adaptation. These results collectively underscore the functional divergence of *LuSOD* genes in mediating stress-specific transcriptional reprogramming, with select members acting as positive regulators of antioxidant defense and others contributing to stress tolerance through negative feedback mechanisms.

### 3.6 Constructing transgenic plants with *LuCSD3* gene and its role in salt stress response

**Figure 10:**
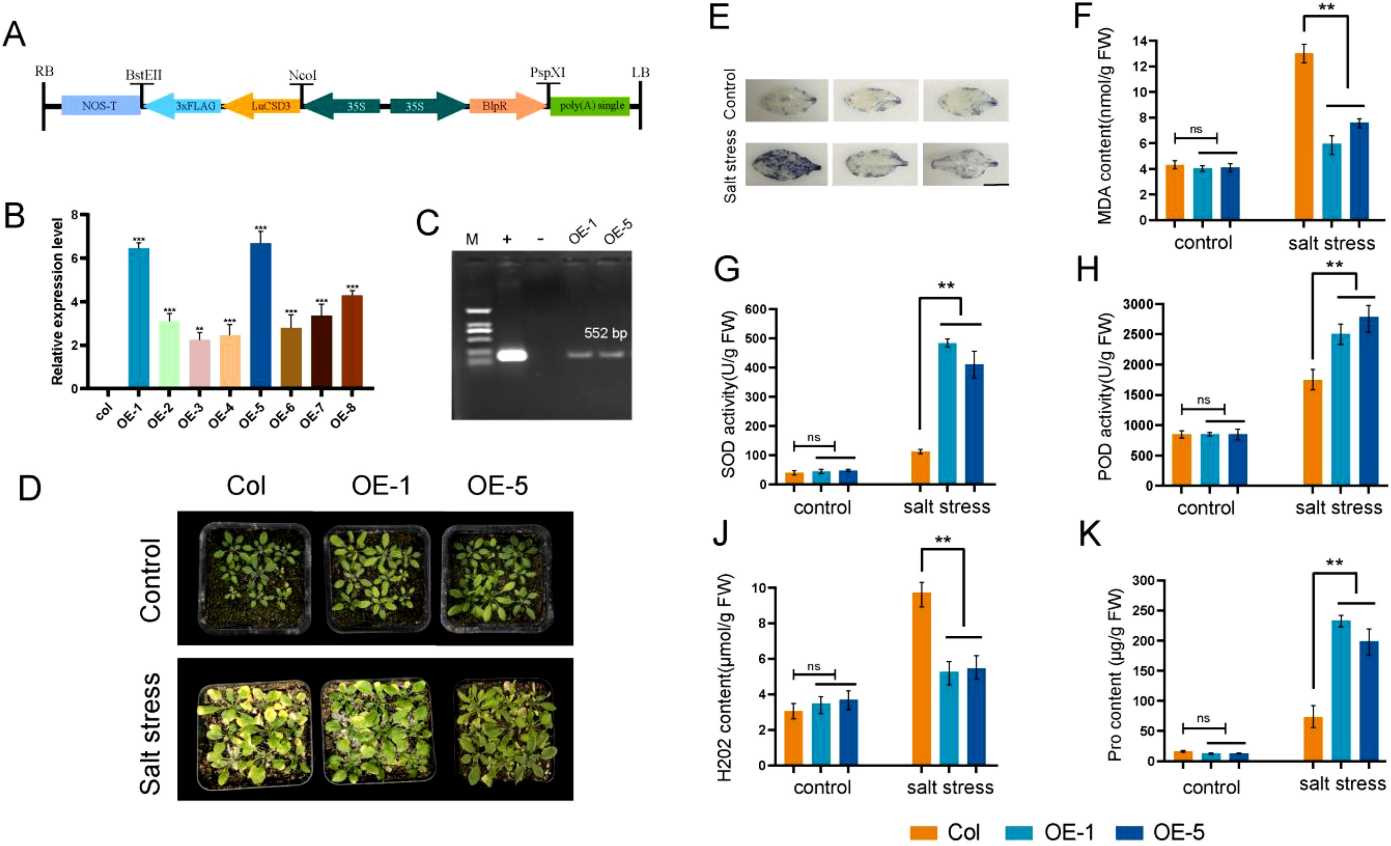
Identification and salt tolerance analysis of *LuCSD3*-overexpressing *Arabidopsis*. **(A)** Schematic representation of the pCAMBIA3301-*LuCSD3* vector. Arrows indicate expression elements, and horizontal lines represent vector restriction sites. **(B)** Gene expression analysis of T3-generation transgenic *LuCSD3 Arabidopsis* lines. **(C)** PCR verification of *LuCSD3* gene expression in 35S::*LuCSD3* overexpression lines (OE-1 and OE-5). M: DNA Marker DL2000; +: recombinant plasmid pCAMBIA3301-*LuCSD3*; -: empty vector pCAMBIA3301 negative control. **(D)** Salt tolerance analysis of overexpression (OE) lines and control (Col) plants. Two-week-old Col and OE plants were watered with or without 200 mM NaCl every three days for 15 days. Photographs were taken to monitor stress phenotypes and measure physiological indices. **(E)** NBT staining of Col and OE leaves under salt stress to detect ROS. Scale bar=1 cm. **(F)** Malondialdehyde (MDA) content. **(G)** Analysis of superoxide dismutase (SOD) activity. **(H)** Peroxidase (POD) activity. **(J)** Hydrngen peroxide (H_2_O_2_) content. **(K)** Proline (Pro) content. Statistical significance of differences is indicated by asterisks: *p < 0.05; **p < 0.01; ***p < 0.001.

Our experimental findings revealed that the *LuCSD3* gene conferred robust stress tolerance in flax. To functionally characterize *LuCSD3*, we engineered a binary expression vector (pCAMBIA3301-*LuCSD3*; **Figure 10A**) and stably transformed it into wild-type *Arabidopsis* (Col-0) via *Agrobacterium*-mediated floral dip. Primary transformants screening identified eight independent T1 lines showing constitutive *LuCSD3* overexpression. Molecular characterization of T3-generation progeny confirmed two homozygous lines (OE-1 and OE-5) with maximal transgene expression levels (**Figures 10B, C**). Salt tolerance tests were conducted on Col and OE lines (OE-1 and OE-5) by exposing plants to 200 mM NaCl stress for 15 days. The results showed that the OE plants exhibited superior growth phenotypes under salt stress, with significantly less wilting compared to the control group (Col) (**Figure 10D**).

### 3.7 Overexpression of *LuCSD3* gene reduces ROS accumulation

To assess the physiological and biochemical impacts of *LuCSD3* overexpression, malondialdehyde (MDA) and proline (Pro) levels were quantified in wild-type (Col) and transgenic lines (OE-1, OE-5) under salt stress (**Figures 10F, K**). Transgenic plants exhibited markedly reduced MDA concentrations and significantly elevated Pro accumulation compared to Col controls. To evaluate *LuCSD3*-mediated regulation of reactive oxygen species (ROS), hydrogen peroxide (H_2_O_2_) content, superoxide dismutase (SOD), and peroxidase (POD) activities were examined. Under non-stress conditions, no statistically significant variations in SOD, POD, or H_2_O_2_ levels were observed between Col and OE lines (**Figures 10G, H, J**). However, salt-stressed OE lines demonstrated a pronounced increase in SOD (1.8-fold) and POD (2.3-fold) enzymatic activities, coupled with a 40% reduction in H_2_O_2_ content relative to controls. These findings were further supported by NBT staining assays, which revealed reduced ROS accumulation in OE leaf tissues under stress (**Figure 10E**). Collectively, these results indicate that *LuCSD3* overexpression enhances salt stress resilience in plants by augmenting antioxidant enzyme activities (SOD, POD) to neutralize ROS and modulating osmoprotectant (Pro) synthesis while minimizing oxidative damage (reduced MDA). This dual mechanism underscores *LuCSD3* as a key regulator of ROS homeostasis under abiotic stress.

### 3.8 Expression pattern analysis of key genes in salt stress pathway in OE-*LuCSD3* strain

**Figure 11:**
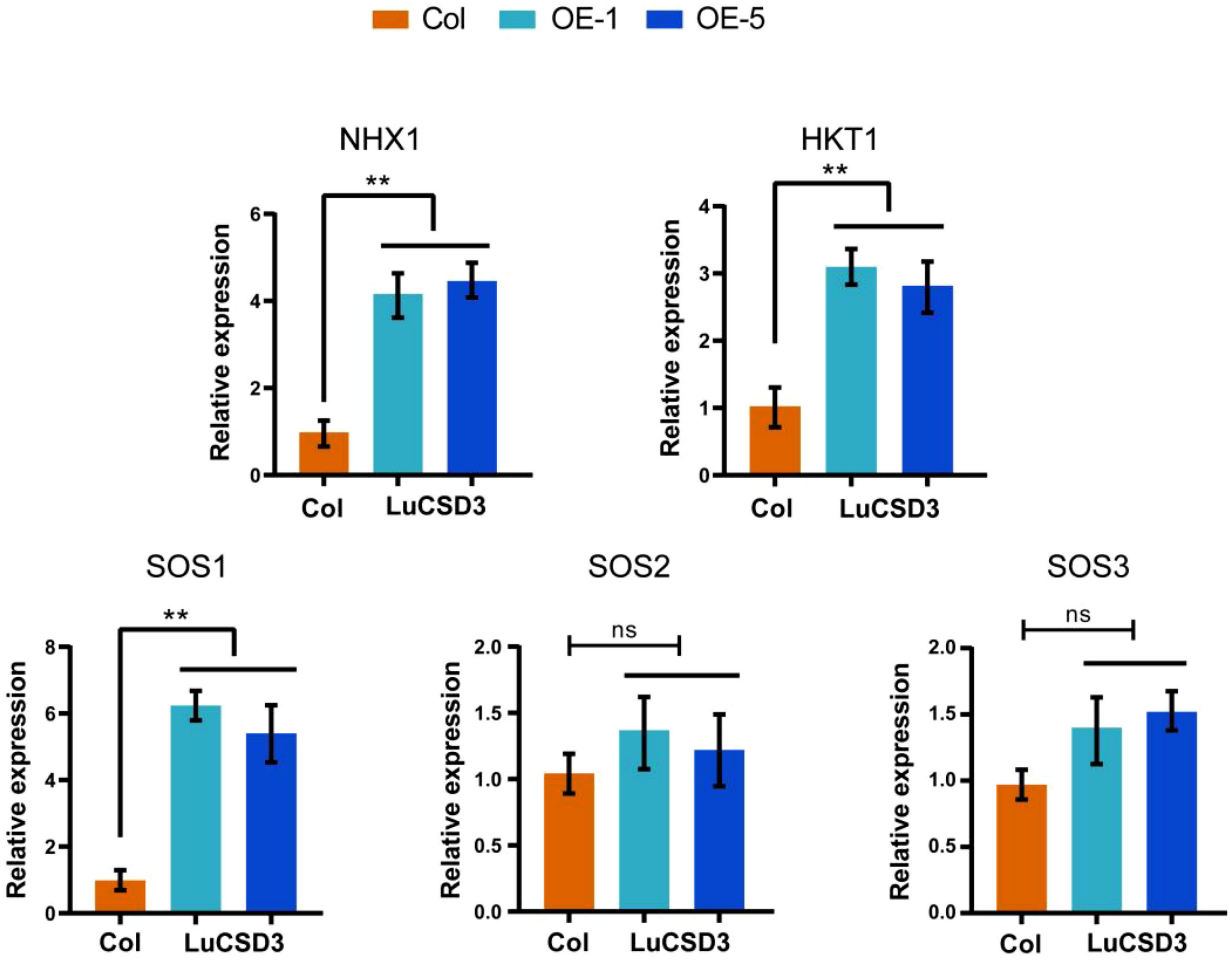
Expression of salt-responsive genes in *LuCSD3*-overexpressing lines. Relative expression levels of *NHX1*, *HKT1*, *SOS1*, *SOS2*, and *SOS3* in transgenic (OE-*LuCSD3*) and wild-type (Col) plants under salt stress. Data are means ± SD (n = 3). Asterisks denote significance (*p < 0.05; **p < 0.01; ***p < 0.001).

When plants encounter salt stress, they employ various mechanisms to resist adverse conditions. The expression levels of five selected salt stress-related genes (*NHX1*, *HKT1*, *SOS1*, *SOS2*, and *SOS3*) were analyzed in OE-*LuCSD3* lines (**Figure 11**). The results indicated that the expression levels of *SOS2* and *SOS3* were not correlated with the expression of *LuCSD3*. However, three key genes (*NHX1*, *HKT1*, and *SOS1*) showed significantly upregulated expression under salt stress. These findings suggest that the *LuCSD3* gene enhances salt tolerance by mitigating ROS accumulation in plants.

## 4 Discussion

Abiotic stress has long been a major constraint on agricultural productivity. Superoxide dismutase (SOD), a critical enzyme in plant stress responses, mitigates oxidative damage caused by salt, drought, and heavy metal toxicity (Gill and Tuteja, 2010). In the past few years, peroxidase family genes in different plants have been identified, such as five peroxidase genes in seaweed (Zang et al., 2020), seven genes in *Medicago truncatula* (Song et al., 2018), seven *SOD* genes in *Rosa chinensis* (Rafique et al., 2023), nine genes in tomato (Feng et al., 2016), 18 genes in cotton (Wang et al., 2017) and 25 genes in banana (Feng et al., 2015), 26 genes were identified in wheat (Jiang et al., 2019), 29 genes were identified in *B. juncea* (Verma et al., 2019), 31 genes were identified in *Brassica napus* (Su et al., 2021). The subfamily distribution of the *LuSOD* genes included six Cu/Zn *SOD* genes, three Fe *SOD* genes, and three Mn *SOD* genes (**Table 1**) (**Figure 1**). This classification aligns with the six *SOD* members reported in *Medicago truncatula* (Song et al., 2018). This relatively moderate number of genes suggested that flax might have relied more on transcriptional and post-transcriptional regulation, rather than extensive gene duplication, to modulate SOD-mediated stress responses. In addition, a large number of studies have shown that *Cu/ZnSOD* gene is mainly distributed in mitochondria, cytoplasmic sol, peroxisome and chloroplast, *FeSOD* gene is mainly distributed in mitochondria and chloroplast, while *MnSOD* gene is almost distributed in mitochondria (Corpas et al., 2006; Wang et al., 2017). This was consistent with the results of this study. The predicted subcellular localizations in the chloroplast, mitochondrion, and cytoplasm emphasized the compartment-specific detoxification of ROS, a well-established strategy for fine-tuning redox signaling under stress conditions (Noctor et al., 2014). Cu/Zn SODs localized to the chloroplast were particularly important in responding to light-induced oxidative stress, while mitochondrial Mn SODs helped alleviate ROS generated during respiration (Alscher et al., 2002).

Analysis of gene architecture demonstrated that the *LuSOD* family in flax exhibited variable exon-intron configurations, with exon numbers ranging from 5 to 11 and introns from 4 to 10 (**Figure 2**). Structurally, the *LuSOD* genes exhibited conserved exon–intron organization and motif composition within each subfamily, reflecting strong purifying selection during evolution. This conservation supported the hypothesis that *SOD* isoforms were functionally constrained due to their critical roles in redox buffering (Fink and Scandalios, 2002). Divergence in *LuSOD* gene organization may reflect evolutionary dynamics driven by exon/intron indels or splicing variations (Xu et al., 2012). These findings align with prior reports on *SOD* gene structure in tomato (Feng et al., 2016) and cotton (Wang et al., 2017), reinforcing the role of structural conservation in stress-responsive gene families.

Gene duplication events played a pivotal role in the evolutionary diversification of *SOD* genes, with repetitive gene copies often driving functional innovation to enhance plant adaptive responses to environmental stressors. Gene duplication of *SOD* genes has also been identified in cotton and rapeseed (Wang et al., 2016; Verma et al., 2019). In this study, eight segmental duplication pairs were detected within the *LuSOD* gene family (**Figure 3A**), indicating that segmental duplication served as the primary driver of *LuSOD* family expansion. Synteny analysis, a robust approach for reconstructing gene evolutionary trajectories, revealed nine collinear ortholog pairs between flax and *Arabidopsis*. These conserved syntenic relationships suggested shared ancestral origins prior to species divergence (Jiao et al., 2014), further underscoring the high evolutionary conservation of *LuSOD* genes in flax.

Cis-regulatory elements within promoter regions orchestrate transcriptional regulation, primarily through sequence-specific interactions between transcription factors and their cognate DNA motifs, a process central to stress-responsive gene expression (Punia et al., 2021). Bioinformatic interrogation of *LuSOD* promoters revealed a predominant enrichment of cis-elements associated with phytohormonal signaling (abscisic acid and methyl jasmonate pathways) and abiotic stress adaptation, including hypoxia response, drought inducibility, and low-temperature tolerance (**Figure 4**). Functional annotation of stress-related motifs identified three core regulatory modules: LTR (low-temperature responsiveness), ARE (anaerobic induction), and TC-rich repeats (drought and defense signaling) (Zhang et al., 2018; Dhatterwal et al., 2021). Notably, the majority of *LuSOD* promoters harbored ARE, LTR, and TC-rich repeat elements, underscoring their conserved role in mediating oxidative stress resilience and environmental adaptation (Osakabe et al., 2014).

MicroRNAs have been recognized as pivotal regulators in harmonizing plant developmental processes and environmental interactions (Song et al., 2019). Our analysis shows that lus-miR159 is the main target miRNA of the *LuSOD* gene family (**Table 2**). In tobacco, miR159-GAMYB pathway plays a role in biological defense response, which is activated after miR159 inhibition (Zheng et al., 2020). In maize, overexpression of miR159 leads to grain enlargement in transgenic plants, indicating that miR159-*ZmMYB* module, as the hub of endosperm development, is involved in endosperm cell division and proliferation (Wang et al., 2023). However, a broader perspective suggested that the SOD–miRNA regulatory module was evolutionarily conserved across plant species and was frequently modulated under abiotic stress conditions. For example, in *Arabidopsis*, miR398 played a central role in oxidative stress signaling by targeting *CSD1* and *CSD2*. Its downregulation under stress led to increased *SOD* expression and enhanced stress tolerance (Beauclair et al., 2010). Comparable modules were reported in cotton, where miR398 regulated *GhCSD1* during salt and drought conditions (Shumayla et al., 2017). These findings highlighted the evolutionary conservation of miRNA-mediated regulation of *SOD* genes across diverse plant taxa. Therefore, the miRNA–*LuSOD* interactions in flax might have represented a broader regulatory circuit involved in stress responses, warranting further experimental validation. Protein-protein interactions were essential not only for maintaining functional integrity but also for predicting functional diversification of proteins (Nobeli et al., 2009). In this study, protein interaction network analysis revealed six *LuSOD* genes with significant pairwise interactions (**Figure 5A**), suggesting their central roles in combating diverse biotic and abiotic stresses. GO enrichment analysis corroborated these findings (**Figure 5B**), aligning with conserved *SOD* functions observed in other crops (Feng et al., 2016; Jiang et al., 2019; Verma et al., 2019). This study underscored the critical role of *LuSOD* genes in flax adaptation to environmental adversities, reinforcing their universal importance in plant stress physiology.

Transcriptomic profiling revealed tissue-specific expression dynamics of *LuSOD* genes across flax organs, with predominant transcriptional activity observed in leaves and floral tissues at 5 and 10 days post-anthesis (**Figures 6B, C**). Salt stress markedly downregulated *LuSOD* expression in leaf and stem tissues, while four genes exhibited upregulated expression in roots and three in leaves under salinity, consistent with prior findings in other species (Su et al., 2021; Yu et al., 2022; Rafique et al., 2023). The ubiquitous expression of these genes across flax tissues suggested their functional involvement in developmental regulation. Under abiotic stress (salt, cold, drought), qRT-PCR analysis of leaf tissues demonstrated significant upregulation of nine *LuSOD* genes, except *LuCSD1*, *LuCSD2*, and *LuFSD3* (**Figures 7**-**9**). Notably, *LuMSD2* and *LuMSD3* exhibited completely opposite transcriptional responses under salt stress compared to cold and drought conditions. *LuMSD2* was significantly downregulated under salt stress but showed strong upregulation in response to cold and drought. In contrast, *LuMSD3* displayed markedly increased expression under salt stress, while its expression was suppressed during cold and drought exposure (**Figures 7**-**9**). This contrasting behavior suggested possible functional divergence despite their shared subcellular localization. Cis-regulatory analysis of their promoter regions revealed distinct features: *LuMSD2* contained a higher abundance of low-temperature and drought-responsive elements such as LTR and MBS, whereas *LuMSD3* was enriched with salinity-related stress-responsive motifs, including ARE and TC-rich repeats (Dhatterwal et al., 2021). The functional divergence might have reflected subtle differences in their roles within mitochondria under specific stress conditions. Under salt stress, mitochondrial metabolism underwent rapid alterations, which might have required a distinct *SOD* isoform to more effectively buffer ROS or to help maintain mitochondrial integrity (Suzuki et al., 2012). *LuMSD3* might have played a more prominent role in acute ROS scavenging, whereas *LuMSD2* might have been involved in slower or regulatory ROS signaling under cold and drought stress conditions, where mitochondrial ROS functioned as secondary messengers (Huang et al., 2019). Comparative analyses across species demonstrated conserved *SOD* upregulation under stress: cucumber *SODs* responded to cold, heat, salt, and drought (Zhou et al., 2017); tomato *SODs* were induced by salt and drought (Feng et al., 2016); wheat SODs showed systemic activation under similar conditions (Jiang et al., 2019). In *Brassica napus*, all *SODs* except *BnCSD6* and *BnFSD1* were stress-inducible, while *Rosa chinensis* roots exhibited elevated *RcCSD1*, *RcCSD3*, and *RcFSD3* expression under salinity (Rafique et al., 2023). Furthermore, this study revealed that the overexpression of the *LuCSD3* gene enhanced the salt stress response in *Arabidopsis*, ultimately improving the plant’s salt tolerance (**Figure 10**). Overexpression of *LuCSD3* in *Arabidopsis* enhanced salinity tolerance by modulating ROS scavenging, analogous to the stress-responsive MeCSOD2 in cassava (Zheng et al., 2023). ROS, critical for cellular homeostasis and signaling, accumulated under stress, inducing oxidative damage (Yang et al., 2015). In this study, transgenic overexpression lines (OE) displayed markedly elevated superoxide dismutase (SOD) and peroxidase (POD) enzymatic activities under salt stress, concurrent with a pronounced reduction in hydrogen peroxide (H_2_O_2_) accumulation. These observations were further validated through nitroblue tetrazolium (NBT) staining assays (**Figure 10**). The *LuCSD3* gene enhances abiotic stress tolerance by upregulating peroxidase and antioxidant enzyme activities in leaves, thereby mitigating ROS accumulation. To elucidate salt adaptation mechanisms, the expression profiles of five well-characterized salt-responsive genes (*AtSOS1*, *AtSOS2*, *AtSOS3*, *AtNHX1*, and *AtHKT1*) were analyzed in both wild-type (Col) and OE lines (Lu et al., 2023; Ma et al., 2023). Compared to the control group, the expression levels of three key genes (*NHX1*, *HKT1*, and *SOS1*) were significantly upregulated under salt stress conditions (**Figure 11**). These findings demonstrated conclusively that *LuCSD3* plays a pivotal role in mediating flax’s response to abiotic stressors. Collectively, this investigation established a framework for deciphering the molecular mechanisms underlying SOD-mediated stress resistance in flax, providing critical insights for future functional studies of the *LuSOD* gene family.

## 5 Conclusions

In this study, the Superoxide Dismutase (SOD) gene family in flax was systematically characterized for the first time. A total of 12 *LuSOD* genes were annotated in the flax genome. Evolutionary relationships, exon-intron architectures, conserved protein motifs, and syntenic patterns were investigated through comparative bioinformatics analyses. miRNA target screening revealed lus-miR159 as the predominant miRNA regulating the *LuSOD* family. Protein interaction networks identified functional linkages among six *LuSOD* members (*LuCSD2*, *LuCSD3*, *LuCSD6*, *LuMSD2*, *LuFSD2*, and *LuFSD3*). GO enrichment highlighted *LuSOD* involvement in stress response pathways, metal ion binding, and enzymatic antioxidant activity. Promoter cis-element profiling demonstrated enrichment of motifs associated with phytohormone signaling (MeJA and ABA) and abiotic stress adaptation. Transcriptomic and qRT-PCR analyses revealed that most *LuSOD* genes exhibited tissue-specific expression patterns, particularly in leaves and floral tissues at 5 days post-anthesis, and responded dynamically to environmental stressors (cold, drought, salt). Functional validation in *Arabidopsis* demonstrated that *LuCSD3* overexpression enhanced salt tolerance by modulating ROS scavenging. Collectively, this comprehensive investigation elucidated the structural and functional diversity of the *LuSOD* family, offering novel insights into SOD-mediated mechanisms underlying flax development and stress resilience.

## Data Availability

The original contributions presented in the study are included in the article/**Supplementary Material**, further inquiries can be directed to the corresponding author/s.
